# Supplementary material for: 3D Polymer Architectures for the Identification of Optimal Dimensions for Cellular Growth of 3D Cellular Models
Source: Polymers (Basel). 2022 Oct 4;14(19):4168. doi: 10.3390/polym14194168 (PMC9572445; doi:10.3390/polym14194168)
Supplement: Supplementary file 1 [file polymers-14-04168-s001.zip › polymers-1906003-supplementary-file S1.pdf]

Matlab code for cell tracking.

```
% ********************** user parameters **********************************
loadFiles = true;       % true / false to load new files
trackCells = true;       % true / false to track cell positions
showCells = true;        % true / false to show the cell positions
N = 5;                   % +/- pixel window size
Ntmin = 4;               % min number of time bins per cell
Ntmax = 6;               % max number of time bins per cell
discardFoundPoints = true;   % discard cell positions once a cell is found
discardControl = true;  % true / false to discard control cells from
analysis
ctrlY = 100;             % y position of the controll scafold (to discard)
showDisplacementMap = true; % true / false show displacement map
dmax = 30;               % maximum displacement (color limit)
% ****************** end of user paramets ********************************

discardFoundPoints = logical(discardFoundPoints + (Ntmin ~= Ntmax)); %
force true if Ntmin ~= Ntmax
if loadFiles
    [FileName,PathName] = uigetfile('*.png','MultiSelect','on'); % file
name and dir
    ims = [];                                  % image stack matrix
    for ii = 1 : length(FileName);             % iterate over selected
files
        ims = cat(3,ims,imread([PathName, '\',FileName{ii}])); %
concatenate loaded files
    end
    ims = double(ims);                         % convert matrix to double
end
figure('color','w')                            % create a new white figure
scaff = (175 < ims(:,:,3)).*(ims(:,:,3) < 185); % find scaffolds in image
cells_raw = ims(:,:,2:3:end) == 160;           % find cells in images
if discardControl
    cells = cells_raw; cells(1:ctrlY,:,:) = 0;  % discard control cells
from analysis
end
[fsy,fsx] = find(scaff);                       % find the scaffold point
plot(fsx,fsy,'.k'); axis image;                % show the scaffold
set(gca,'ydir','reverse');                     % show the image in the
typical direction
%imagesc(scaff); axis image; colormap(flipud(gray)) % show the scafold
hold on
if trackCells
    ds = [];                                   % array of cell
displacements
    dmap = nan(size(cells,1),size(cells,2));    % map of cell displacements
    for nt = Ntmax:-1:Ntmin                    % iterate over min number
of time bins / cell
        for ii = N+1 : (size(cells,1) - N)      % iterate over y
            for jj = N+1 : (size(cells,2) - N)  % iterate over x
                subcells = cells(ii-N : ii+N , jj-N : jj+N,:);  % find
[2N+1,2N+1] window
                if sum(subcells(:)) == nt       % number of points matches
the time bin requirements
                    xs = zeros(1,nt);           % x array of cell positions
```

```
                    ys = xs;                    % y array of cell positions
                    nCells = 0;                 % number of cells per time
slice
                    fail = false;           % in case no cell is found
                    for kk = 1 : nt             % iterate over the time
slices
                        [ya,xa] = find(subcells(:,:,kk));   % find cell
positions
                        nCells = max(nCells,length(xa));    % count number
of cells per slice
                        if (nCells == 1) && ~isempty(xa) % if only 1 cell
per slice
                            try
                                xs(kk) = xa + jj - N - 1;   % store cell
trajectory
                                ys(kk) = ya + ii - N - 1;   % store cell
trajectory
                            catch
                                fail = true;    % in case no cell is found
                            end
                        else
                            fail = true;
                        end
                    end
                    if (nCells == 1) && ~isempty(xa) && ~fail  % one and
only one cell is found
                        d = sum(sqrt(diff(xs).^2 + diff(ys).^2)); % total
displacement
                        ds(end+1) = d;          % store in displacements
array
                        dmap(round(mean(ys)),round(mean(xs))) = d;        %
store in displacement mp
                        plot(xs,ys,'linewidth',2,'color',rand(1,3))   %
plot the cell trajectory
                        if discardFoundPoints
                            cells(ii-N : ii+N , jj-N : jj+N,:) = 0; %
discard already counted cells
                        end
                    end
                end
            end
        end
    end
end

if showCells
    cmap = jet(size(cells,3));                  % assign different colors
to the time bins
    for ii = 1 : size(cells,3)
        [y,x] = find(cells_raw(:,:,ii));            % find the cells in
each time bin
        plot(x,y,'o','color',cmap(ii,:),'linewidth',2)  % show the cells
    end
end

if showDisplacementMap
    c = imagesc(inpaint_nans(dmap,5));
    uistack(c,'bottom');
    colormap(jet(255))
    caxis([0 dmax])
end
```
